# Refugees have cancer too

**DOI:** 10.3332/ecancer.2024.ed134

**Published:** 2024-06-27

**Authors:** Mona Ali Hassan, Akash Maniam

**Affiliations:** Portsmouth Hospital University NHS Trust, Portsmouth PO63LY, UK

**Keywords:** cancer care, refugees, war, conflict areas

## Abstract

Managing cancer under ideal conditions is a daunting prospect, to say the least. Treating cancer in conflict areas, war zones or being a refugee with cancer, facing complex political, economic and health-related threats presents a colossal global challenge. Managing such patients requires close coordination with international bodies, nongovernmental organisations and national governments, mitigating the burden of cancer care provision to patients and host countries alike.

Managing cancer in modern times requires a high degree of resources and flexibility. Cancer diagnosis worldwide has been increasing significantly with predictions that it will be one of the most common diseases in the future and across the world. In normal circumstances there is a big gap in access to cancer care between developing and developed countries where patients in developed countries have unparalleled access to trials, diagnostics and therapeutics, all resulting in higher survival rates. This mandates efficiency and close coordination among healthcare providers, centers and thorough discussions with patients to ensure appropriate decision-making. Handling this is an enormous challenge globally, even in well-resourced settings. This difficulty increases drastically in times of war, both domestically and internationally, as providing cancer care to refugees is a uniquely complicated task.

Unfortunately, with active wars in Gaza, Sudan and Ukraine, we are witnessing increased numbers of refugee cancer patients without access to any form of health care. In Gaza, the sole cancer center was bombed, threatening the lives of 10,000 cancer patients 1. According to Alrawa et al 2, the Sudanese military conflict on 15 April 2023, had a profound impact on cancer care, likewise in Ukraine where Russia’s ongoing war has led to substantial delays in cancer treatment, both due to displacement and widespread bombardment of healthcare facilities and is estimated to cause an increase in cancer-related death by 3,600 3.

Furthermore, due to earlier wars, most notably in the Middle East, there are vast numbers of refugees worldwide. To wit, while Afghanistan, Iraq, Syria and Yemen still grapple with the lasting impacts of war, the incidence of cancer has risen but with scarce cancer care access at best.

Cancer patients in such conflict areas must not only survive war against aggression but also battle against diseases that, if left untreated, lead to death and morbidity. Moreover, refugee patients face all the challenges that come with their status, including impaired access to basic needs, viz. shelter, clean water and food. Additionally, they are particularly vulnerable to outbreaks of communicable diseases, while lacking access to their treatment, both due to pharmaceutical supply disruption and compromised hospital function because of fuel, personnel or infrastructural shortages. Tragically, drug shortages often most harshly impact palliative patients needing opioid analgesics for cancer-associated pain, who consequently face immense suffering with most patients ending up with disease progression because of inability to access care and not being able to access any kind of treatment such as radiotherapy, chemotherapy, immunotherapy or even hormonal therapy. Also, a big part that is affected is palliaitive care and access to pain medication which we all know is very important to any cancer patient [4]. Thus, even basic cancer and supportive care is often impossible.

Outside of conflict zones, cancer poses a massive burden for both refugees and host countries especially since sometimes the other part of their country or the different country that they seek refuge in does not have the capacity to manage such patients in there health system which also creates pressure on hosting areas. Understandably, under wartime conditions, international organisations and medical nongovernmental organisations (NGOs) focus chiefly on providing urgent medical care for the wounded and primary medical care on communicable diseases for refugees, such as cholera, as they present more immediate threats to life. However, cancer is no less dangerous.

In Gaza, for example, there are around 10,000 cancer patients part of them were evacuated to Turkey, Egypt, UAE or Jordan, while adult patients were transferred to Egypt, Turkey and the UAE to continue their treatment. This is not a sustainable strategy, nor is it desirable, as it further displaces vulnerable patients from critical familial, cultural and social support structures. Switching to oral treatments where applicable and focusing on the best supportive care are options to help decrease the burden on both cancer patients and countries facing war and displacement, but again these are temporary emergency measures, not long-term goals. These also further widen outcome disparities in cancer care between resource-deprived and resource-rich countries.

In conclusion, unfortunately, in the current geopolitical climate, we are at an ever-increasing risk of having to confront such challenges, which highlights the importance of preparing contingency plans, ideally in coordination with international organisations. This would allow the management of such patients even amidst war and displacement. Fundamentally, refugees are human beings who did not choose these circumstances and deserve equitable access to treatment, whether cancer-related or not. Being a refugee or at war neither prevents nor shields oneself against cancer and although some argue that this ought not to be a wartime priority, every life is vital and we must save as many as possible.

## Conflicts of interest

The authors declare no conflicts of interest.

## Funding

No funding was available for this article.
